# Best management modality of trichobezoar: A case report

**DOI:** 10.1016/j.ijscr.2018.11.030

**Published:** 2018-11-22

**Authors:** Emad M. Al-Osail, N.Y. Zakary, Yasir Abdelhadi

**Affiliations:** a Department of General Surgery, College of Medicine, Imam Abdulrahman Bin Faisal University, Dammam, Saudi Arabia; b King Fahad Military Medical Complex, Dharan, Saudi Arabia

**Keywords:** Trichobezoar, Endoscopy, Laparotomy, Case report, Psychiatric

## Abstract

**Introduction:**

Trichobezoar is a rare condition, usually diagnosed in children and young female especially with psychiatric illness. Diagnosis of trichbezoar is usually dependent on the disease history, examination, diagnostic modality including CT scan. Generally this condition can be treated by endoscopy, laparoscopy or laparotomy. Here, we have presented a rare case of trichobezoar which has failed to be treated endoscopically but was eventually treated successfully by laparotomy.

**Case report:**

A 17 years old girl was presented with the initial complaint of epigastric pain for about 9 months duration with symptoms including vomiting and weight loss. Clinical examination showed tenderness and hard mass over epigastric area and it was extending towards the right hypochondrial area. CT scan results also showed that the stomach was filled with large intra-luminal abnormal mass like contents with mottled air pattern extended to fill the pylorus and first part of the duodenum. These findings were the proofs for possible trichobezoar. Endoscopic surgery failed significantly and hence laparotomy was performed, as a result, trichbezoar was removed successfully.

**Discussion:**

Trichobezoar can be treated by using endoscopy for removal of hair, laparoscopy or Laparotomy. Laparotomy had 100% successful rate while 5% for endoscopy and 75% for laparoscopy.

**Conclusion:**

Laparotomy is the best treatment method for trichobezoare. Psychiatric management should be done for any patient with trichotillomania to avoid trichobezoar.

## Highlights

• Laparotomy is the best treatment method for trichobezoare.
• Psychiatric management should be done for any patient with trichotillomania to avoid trichobezoar.
• Endoscopic treatment can only be successful for small trichobezoar.

## 1 Background

The term “bezoar” is referred as any partially digested or undigested foreign material trapped inside the gastrointestinal tract (GIT) [1]. The classification of bezoar depend on their composition and are usually of four types:1) Phytobezoare, composed of indigestible vegetable fibers and are deposited at GIT, 3) Lactobezoar: composed of milk proteins and mucus, 3) Trichobezoar: which is composed of hair in the stomach, 4) Pharmacobezoars which is composed accumulated dissolved medications or drugs and there is another unclassified bezoars which is accumulation of any other type of materials in GIT other than the above mentioned components [2]. Trichobezoar is a rare entity, usually seen in children and young female especially with psychiatric illness [3]. Trichobezoar was first identified in 1779 by Baudomant [4]. Stomach has smooth surface and it resists the digestion and peristalsis of human hair, but if the person continuous to ingest the hair, it will lead to accumulation of hair with mucus and food leading to trichobezoar. It is usually found in the stomach but can also be observed at small intestine or colon and this is known as Rapunzel syndrome, described in 1968 by Vaughan et al. [5]. Diagnosis of trichbezoar usually depend on the disease history, examination, diagnostic modality such as computed tomography (CT) scan and can be treated by endoscopy, laparoscopy or laparotomy. Here we have presented a rare case of trichobezoar which failed to get manage through endoscopy and eventually treated successfully by laparotomy. This work has been reported in accordance with the SCARE criteria [6].

## 2 Case presentation

### 2.1 Patient information

A 17 years old girl was admitted to the emergency department (ED) with history of epigastric pain for 9 months duration along with severe symptoms of vomiting (undigested food and bloody content), drastic weight loss (around 4 kg over 4 months). Patient was not having any urinary symptom or any altered bowel habit.

-Past medical history: Instances of eating hair years ago.

-And no history of previous surgery.

### 2.2 Clinical finding

-On Examination:

Temperature: 36.8 °C, Pulse rate: 125 beat per minute, Breathing rate: 22 breaths per minute and Blood pressure: 119/65 mmHg.

Abdomen: There was tenderness and hard mass over epigastria area extending towards the right hypochondrial area. Bowel sound was sluggish.

### 2.3 Diagnostic assessment

#### 2.3.1 Laboratory studies

CBC, LFT, RFT was within normal limit.

#### 2.3.2 Radiological imaging

CT scan of abdomen was performed at another Hospital: The stomach was grossly distended, compressed with displacement of the transverse colon. The colon was filled of intra-luminal abnormal mass like contents with mottled air pattern extended to fill the pylorus and first part of duodenum. Findings were the proofs for possible trichobezoar.

#### 2.3.3 Therapeutic intervention

Endoscopic removal of hair was failed and the patient was prepared for the operation.

**Fig. 1:**
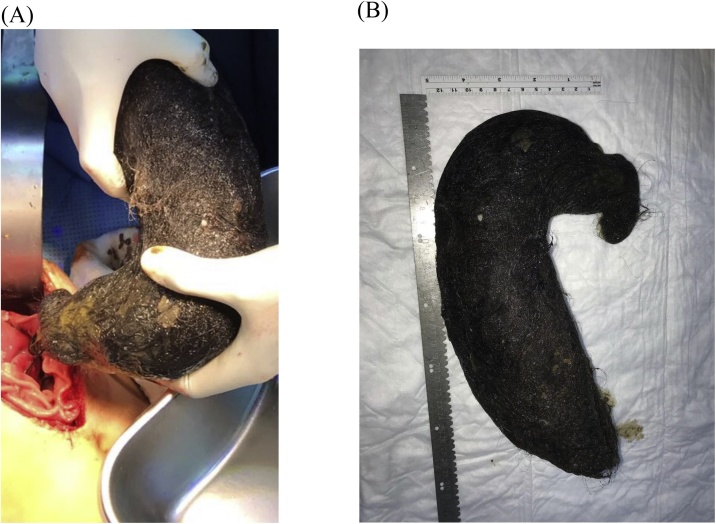
A and B: showed trichobezoar take the shape of stomach.

Upper midline incision was performed and was deepened using electrocautery. After entering the abdominal cavity, distended stomach with firm content was observed. Vertical gastrostomy was done using electrocautery of about 10–14 cm in length. The stomach was found with full of ingested hair, occupying most of the stomach. Ball of hair were then retrieved and removed (Fig. 1A and B). Suction and irrigation were done. Negative suction tube was inserted and the position was adjusted intra-operatively. The stomach was then closed in two layers at continuous manner. First layer was closed with full-thickness using 3-0 PDS and the other layer with Lembert technique using 3-0 PDS. Then the small bowel series or test relatively collapsed with no evidence of any distal obstruction. After suction and irrigation, homeostasis was achieved and then the abdominal wall was closed by using Loop PDS. Staplers were used in surgery in place of sutures to close the skin. The patient tolerated the procedure, recovered smoothly and then was sent to the recovery room with general stable condition.

#### 2.3.4 Follow up

Day 4: Post operation nasogastric tube (NGT) was removed and on the next day, the patient was discharged with outpatient clinic follow up (next week in 07/06/2017).

In clinic, the surgical wound was found to be infected with pus, but there was no sign of cellulites and dressing was done.

13/06/2017: Vacuum (VAC) dressing was applied.

22/06/2017: The wound was cleaned and VAC dressing was removed.

31/07/2017: Patient had an appointment at the psychiatric clinic and she was found stable with no active psychiatric complains.

20/09/2017: The wound was completely healed and the patient was discharged from the General Surgery Clinic.

## 3 Discussion

Trichobezoar is a rare condition which can be asymptomatic at early stages. It is very common among young females especially between 10–19 years old, who have previous history of psychotic diseases such as trichotillomania (the patient pulls out her own hair) and trichophagia (the patient swallows her own hair). It can be also associated with some other psychiatric diseases such as pica, obsessive compulsive disorder, depression, anorexia nervosa and mental diseases [[7], [8], [9]]. If the diagnosis is not done at the early stage, trichobezoar continuously grows leading to the erosion of gastric mucosa, causing ulcers, perforation, intussusceptions, obstructive jaundice, enteropathy due to protein lose, pancreatitis and death. All these conditions have been reveled in the literature [[10], [11], [12], [13], [14]].

The severity of trichobezoar depends on the degree of obstruction. Symptomatic patient usually shows symptoms like abdominal pain, abdominal mass nausea, bilious vomiting, anorexia, hematemesis, weight loss, early satiety, weakness and weight loss. These are also the signs of acute abdomen condition and intestinal obstruction [7].

Radiological imaging is used in trichobezoar diagnosis. In this case report the Ultrasound (U/S) of abdomen have shown the presence gastric mass. Generally, CT scan of abdomen is better than U/S to detect trichobezoar, but the best diagnostic tool considered for detection is endoscopy [7,8,15].

Trichobezoar can be treated by using endoscopy for removal of hair, laparoscopy or laparotomy. In the literature endoscopic treatment of trichobezoar have shown low successful rate, with around 40 cases of trichobezoar tried to be treated through endoscopy, only two case were treated successfully around (5%). Endoscopic treatment can only be successful for small trichobezoar [16,17]. Repetitive attempt of endoscopic treatment can lead to pressure ulcers, esophagitis and esophageal perforation [18,19].

Laparoscopy can be another option for treatment of trichobazoar. First case report of trichobezoar treated with laparoscopy was published by Nirasawa et al. After that laparoscopic method was performed in six cases, where two cases were failed to be treated with laparoscopy, while the rest were successful [20].

Laparotomy is considered to be the best method for treatment of trichobezoar. Retrospective study of 108 patients diagnosed with trichobezoar was performed by Gorter et al. The study concluded that the success rate of endoscopic treatment was 5%, while 75% for laparoscopic treatment. And 100% success rate was achieved by laparotomy [21].

In case of Rapunzel syndrome, the large amount of hair extends beyond the stomach, and hence endoscopic fragmentation treatment usually fails and it shouldn’t be performed. Under these conditions, laparotomy must always be preffered [22,23].

In this study, the patient was a young female with known history of trichotillomania not evaluated by psychiatry. Patient was presented with classical symptoms and signs of trichobezore. Endoscopic treatment failed and trichobezoar was treated successfully by laparotomy.

**In summary**, endoscopy should be used only as a diagnostic method for trichobezoar while laparotomy surgery should be done for any patient diagnosed with trichobezoar. Patient ones had trichotillomania should be evaluated and treated psychologically to avoid chances of trichobezoar and the investigation should be done at the earliest.

## Conflicts of interests

The authors denies any conflict of interest.

## Funding source

There are no sponsors and there was no special funding for writing or publication of this case report.

## Ethics approval

Approval has been granted by the Clinical Research Committee based on written consent from the patient.

## Consent

Written consent was taken from the patient for publication of this case report and the accompanying images.

## Author contribution

Emad M. AL-Osail: Wrote the Manuscript.

N.Y. Zakary: Did Endoscopy.

Yasir Abdelhadi: Review the manuscript.

## Registration of research studies

N/A.

## Guarantor

Emad M. AL-Osail and Yasir Abdelhadi.

## Availability of data and materials

The datasets used during the current study are available from the corresponding author on reasonable request.

## Provenance and peer review

Not commissioned, externally peer reviewed.
